# Corpora amylacea are associated with tau burden and cognitive status in Alzheimer’s disease

**DOI:** 10.1186/s40478-022-01409-5

**Published:** 2022-08-08

**Authors:** Connor M. Wander, Tamy Harumy Moraes Tsujimoto, John F. Ervin, Chanung Wang, Spencer M. Maranto, Vanya Bhat, Julian D. Dallmeier, Shih-Hsiu Jerry Wang, Feng-Chang Lin, William K. Scott, David M. Holtzman, Todd J. Cohen

**Affiliations:** 1 Department of Neurology, UNC Neuroscience Center, University of North Carolina at Chapel Hill, Chapel Hill, NC USA; 2 Department of Pharmacology, University of North Carolina, Chapel Hill, NC USA; 3 Department of Biostatistics, University of North Carolina, Chapel Hill, NC USA; 4 Bryan Brain Bank, Department of Neurology, Duke University School of Medicine, Durham, NC USA; 5 Department of Neurology, Hope Center for Neurological Disorders, Knight Alzheimer’s Disease Research Center, Washington University School of Medicine, St. Louis, MO USA; 6 Brain Endowment Bank, Department of Neurology, University of Miami Miller School of Medicine, Miami, FL USA; 7 Department of Pathology, Duke University School of Medicine, Durham, NC USA; 8 John P. Hussman Institute for Human Genomics, University of Miami Miller School of Medicine, Miami, FL USA; 9 Dr. John T. Macdonald Foundation Department of Human Genetics, University of Miami Miller School of Medicine, Miami, FL USA; 10 Department of Biochemistry and Biophysics, University of North Carolina, Chapel Hill, NC USA

## Abstract

Corpora amylacea (CA) and their murine analogs, periodic acid Schiff (PAS) granules, are age-related, carbohydrate-rich structures that serve as waste repositories for aggregated proteins, damaged cellular organelles, and other cellular debris. The structure, morphology, and suspected functions of CA in the brain imply disease relevance. Despite this, the link between CA and age-related neurodegenerative diseases, particularly Alzheimer’s disease (AD), remains poorly defined. We performed a neuropathological analysis of mouse PAS granules and human CA and correlated these findings with AD progression. Increased PAS granule density was observed in symptomatic tau transgenic mice and APOE knock-in mice. Using a cohort of postmortem AD brain samples, we examined CA in cognitively normal and dementia patients across Braak stages with varying APOE status. We identified a Braak-stage dependent bimodal distribution of CA in the dentate gyrus, with CA accumulating and peaking by Braak stages II–III, then steadily declining with increasing tau burden. Refined analysis revealed an association of CA levels with both cognition and APOE status. Finally, tau was detected in whole CA present in human patient cerebrospinal fluid, highlighting CA-tau as a plausible prodromal AD biomarker. Our study connects hallmarks of the aging brain with the emergence of AD pathology and suggests that CA may act as a compensatory factor that becomes depleted with advancing tau burden.

**Supplementary Information:**

The online version contains supplementary material available at 10.1186/s40478-022-01409-5.

## Introduction

Corpora amylacea (CA) are spherical structures ranging from approximately 10 to 50 µm in diameter that are present in the central nervous system [1–3] and other tissues (e.g., liver, prostate, skeletal muscle) where they serve as putative waste disposal sites referred to as wasteosomes [4]. CA are densely packed and organized in a concentric ring structure, primarily composed of carbohydrates [5], but can contain cellular macromolecules, proteins, and even organelles within this dense matrix, [6–8]. Periodic acid-Schiff (PAS) granules are considered the murine analogs of CA, however PAS granules are typically smaller (1–5 µm) and their exact molecular composition and elimination pathways are not well understood [2]. Insights into the putative role of human CA can be gleaned from prior analysis of PAS granules in mice [9]. For example, PAS granule density increases in response to neuroinflammatory stimuli including cerebral infection [10], lipopolysaccharide (LPS) [11], oxidative stress [12], familial Alzheimer’s disease (AD) transgene expression [13], and natural aging [11, 14, 15], suggesting a protective role in maintaining normal brain homeostasis [16]. We previously analyzed PAS granules in an aged panel of mice and found them localized as distinct clusters in the hippocampus of middle-aged (~ 12 months) and older mice (> 24 months) but were nearly undetectable in young wild-type mice (< 6 months) [17]. Moreover, we found PAS granule density was elevated in early-stage triple transgenic 3xTg-AD mouse brain harboring both tau and beta-amyloid (Aβ) pathology.

How CA are involved in aging or age-related neurodegeneration is unclear. In our prior study, we considered that CA might harbor early-stage tau species that has evaded prior detection but was implied via mass spectrometry analysis of CA from human AD brain tissue [18]. Using a series of highly specific immunofluorescence approaches, we detected tau within mouse PAS granules and human CA (CA-tau) [17], which had distinct molecular and biochemical properties compared to classic end-stage tau pathology. For example, CA-tau is immunoreactive with the non-phosphorylation dependent Tau-1 antibody (indicating hypo-phosphorylation at this epitope), which was validated by reduced immunoreactivity at the AT8 epitope (S202/T205) [19]. This indicates that CA-tau is less detectable with AT8, the standard tau antibody used for AD diagnostics. Staining CA with amyloid-binding dyes (i.e., thioflavin-S, congo red) that typically label the β-structure of mature neuro-fibrillary tangles (NFTs) has produced inconsistent results [20–22], suggesting possible heterogeneity of CA cargo. These unique histological properties warranted a more thorough evaluation of CA in AD brain tissue [2].

Our initial postmortem analysis of human control and AD brain suggested tau-positive CA may decline in AD brain, an indicator of reduced neuroprotection through gradual CA depletion [17]. Here, we sought to establish a link between CA/PAS granule formation and accumulating tau burden in mice and human brain. We identified accelerated PAS granule formation in tau transgenic and APOE mouse models of AD pathology, which prompted a detailed evaluation of CA in a large panel of human AD brains. With advancing Braak stage, we report a bimodal distribution of CA in the hippocampus of AD patients. These findings highlight CA and their tau cargo as a new modality to evaluate brain resilience and as a potential biomarker for AD onset or progression.

## Results

### PAS granules in tauopathy and APOE mice

We previously reported that the formation of PAS granules, the murine analogs of CA, was accelerated in 6-month-old 3xTg-AD mice when compared to controls, with their density peaking at approximately 10 months and gradually plateauing until 24 months [17]. 3xTg-AD mice harbor both human Aβ and tau transgenes; however, even at an advanced age, they do not display overt neuronal loss fully resembling AD-related neurodegeneration [23]. PAS granules are also elevated in a humanized model of Aβ pathology [24], however, no reports have investigated PAS granule dynamics in a symptomatic tauopathy model of neurodegeneration, in which tau pathology recapitulates advancing Braak stage in human AD.

**Fig. 1:**
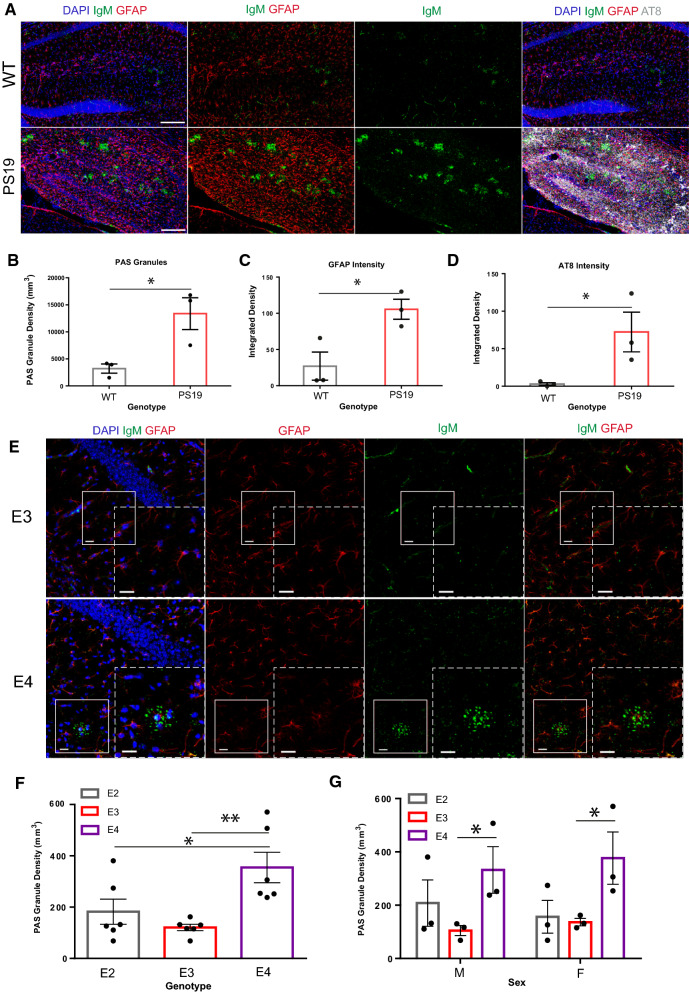
PAS granule density in tauopathy and AD risk mouse models. **A** Representative immunofluorescent confocal images of 8–11-month-old wild-type control and age matched PS19 hippocampal sections stained with GFAP (red), AT8 (grey), and IgM (green). Nuclei labeled with DAPI. 40 µm thick coronal sections, scale bar = 250 µm. **B** Comparison of PAS granule density (mm^3^) in 8–11-month-old wild-type control and age-matched PS19 hippocampal sections. *P* = 0.0292. **C** Comparison of GFAP intensity in 8–11-month-old wild-type control and aged matched PS19 hippocampal sections. P = 0.030. **D** Comparison of AT8 intensity in aged wild-type control and PS19 mice. p = 0.0296. **E** Representative immunofluorescent images of hippocampal sections from 6-month-old E3 and E4 mice stained with GFAP (red) and IgM (green). Nuclei labeled with DAPI (blue). 40 µm thick coronal sections, scale bar = 20 µm. **F** Comparison of PAS granule density (mm^3^) in pooled 6-month-old *APOE* E2, E3, and E4 mice. *p = 0.0492, **p = 0.0033. **G** Comparison of PAS granule density (mm^3^) in male and female 6-month-old *APOE* E2, E3, and E4 mice. *p = 0.0426 (male), 0.0343 (female)

We analyzed PAS granule biogenesis in PS19 mice overexpressing 1N4R human tau harboring the P301S mutation, which generates hyperphosphorylated and thioflavin-positive tau aggregates, leading to neuronal atrophy and cognitive deficits [25, 26]. Hippocampal sections from 7 to 9 month-old PS19 and age-matched littermates were analyzed with antibodies that detect astrocytes (GFAP), phosphorylated tau (AT8), and a carbohydrate-rich neoepitope within PAS granules (IgM). The exogenously applied IgM antibody isotype is recruited to PAS granules and therefore serves as a robust PAS granule marker (Fig. 1A) [27, 28]. Quantification of confocal images showed that PS19 mice harbored significantly more hippocampal PAS granule density when compared to non-transgenic littermate controls (Fig. 1B), which paralleled the hallmark signs of astrogliosis (Fig. 1C) and tau pathology (Fig. 1D) [29].

Considering both Aβ and tau pathology are sufficient to induce PAS granule formation, we next considered whether these structures correlated with APOE status, the single most prominent genetic risk factor for AD. We considered this possibility because PAS granules and CA are known to be closely associated with and likely generated within astrocytes [30, 31], cells that are increasingly reactive in neurodegenerative diseases [32], naturally aged mice [33], and human *APOE* E4 knock-in (KI) models [34]. We therefore assessed differences in PAS granule formation in an APOE mouse model using a recently developed humanized line in which the mouse *APOE* locus was replaced with human *APOE* E2, E3, or E4 (APOE-KI mice) and subsequently evaluated PAS granule formation in 6-month old animals [35]. Brain sections were labeled with GFAP and IgM antibodies to detect both astrocytes and PAS granules (Fig. 1E). Quantification of immunofluorescence images showed increased PAS granule density in *APOE* E4 compared to *APOE* E3 mice (Fig. 1F). Female *APOE* E2 mice also harbored significantly fewer PAS granules than female *APOE* E4 mice, a difference that was not similarly observed in males (Fig. 1G). Overall, these data support the notion that PAS granule accumulation can be linked to both AD risk and accumulation of AD pathology in mice.

### Bimodal CA density in AD

**Fig. 2:**
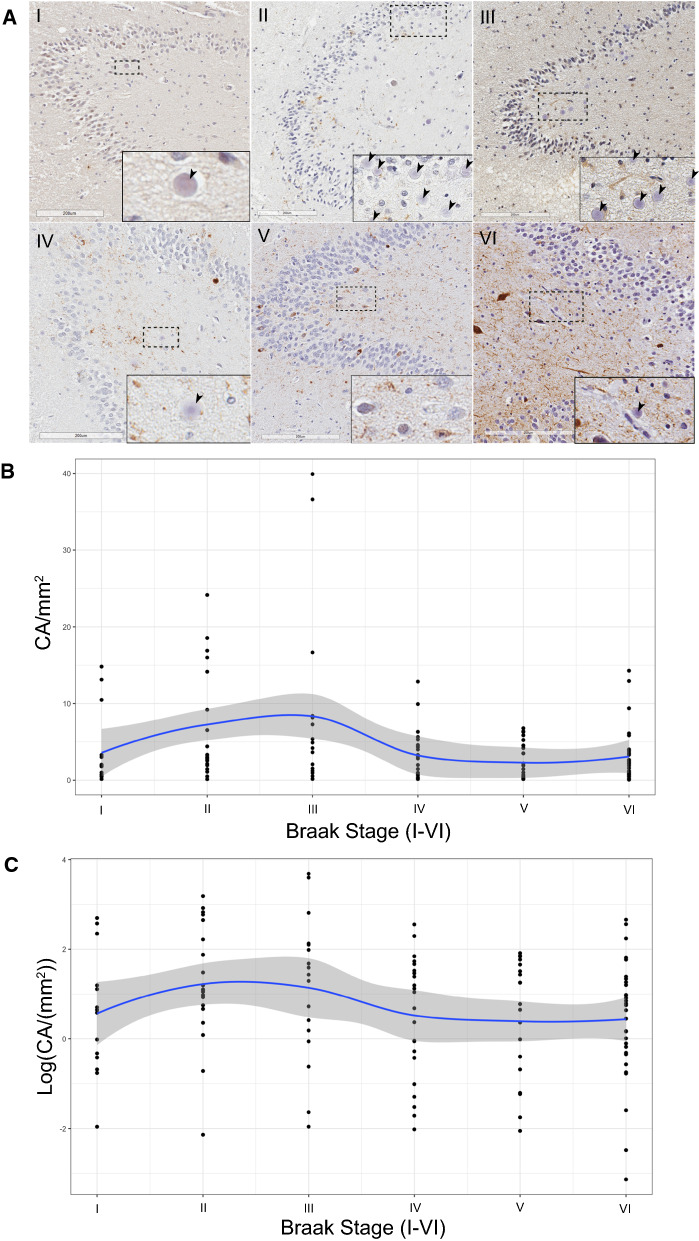
Bimodal CA density in AD brain. **A** Brightfield scans of immunostained AT8^+^ human postmortem tissue from patients with increasing Braak stages (I–VI). Inset denotes representative images of spherical CA, which are denoted by black arrowheads, when present. Scale bar = 200uM. **B** Scatter plot of patient CA/mm^2^ vs Braak stage (I–VI), linear scale. Blue line denotes mean, grey line denotes interquartile range. **C** Scatter plot of patient CA/mm^2^ vs Braak stage (I–VI), logarithmic scale. Blue line denotes mean, grey cloud denotes interquartile range

We next sought to determine whether CA follow similarly distinct patterns in human brain. To determine CA differences in human brain as a function of cognitive decline and AD progression, we analyzed hippocampal tissue from 124 brains spanning Braak stages I-VI. The descriptions of all patient characteristics are shown in Table 1. We focused our CA analysis exclusively on the hippocampus for two main reasons. First, hippocampal volume loss is associated with severity of AD pathology and cognitive deficits [36]. Second, the hippocampus is a focal point for tau spread into higher neocortical regions and a putative zone of CA extrusion into the ventricles and CSF [16]. A minimum of n = 10 cases at each Braak stage (I-VI scale) were immunostained with anti-phosphorylated tau (AT8) and counterstained with hematoxylin to mark CA while distinguishing them from nuclei (Fig. 2A). We selected and scanned the dentate gyrus (DG) as our main region of interest in proximity to the hippocampal sulcus (Additional file 1: Figure S1A). The analysis of CA abundance, as determined by CA counts per mm^2^, showed a skewed distribution that was partially normalized using a logarithmic scale (Additional file 2: Fig. S1B, C).

**Table 1:** Sample characteristics describing distributions of major covariates encompassing n = 124 hippocampal patient tissue samples in this study. Extended sample characteristics available in Table 4.

Characteristic	N = 124
*APOE allele*	
E2/E3	2 (1.6%)
E3/E3	87 (70%)
E3/E4	28 (23%)
E4/E4	7 (5.6%)
*Age (years)*	
Mean (SD)	79 (13)
Median (IQR)	81 (69, 90)
Range	43, 101
*Sex*	
F	65 (52%)
M	59 (48%)
*Ethnicity*	
AA	2 (1.6%)
Mult	1 (0.8%)
W	121 (98%)
*Cognitive status*	
Cognitively normal	32 (26%)
Mild impairment	7 (5.7%)
Dementia	84 (68%)
Not recorded	1
*Braak stage (I–VI)*	
I	14 (11%)
II	21 (17%)
III	17 (14%)
IV	23 (19%)
V	19 (15%)
VI	30 (24%)

When stratifying patients based on Braak stage, the data revealed a bimodal CA distribution, in which CA were sparse at early Braak (I) and late Braak stages (V-VI), with a peak at Braak II-III (Fig. 2B, C, and see box plot in Additional file 2: Fig. S1D). To further define the association between CA and tau burden, we employed a generalized linear model controlling for covariates of age, sex, postmortem interval (PMI), APOE status, and cognitive status (Table 2). The generalized linear model, fitted with a gamma distribution and logarithmic link function (Fig. 2C, see box plot in Additional file 2: Fig. S1E), detected a significant relationship between Braak stage and CA abundance (*p* = 0.006). Multiple comparisons using the Bonferroni method detected a significant difference in CA abundance between Braak III and V (*p* = 0.034) (Table 3).

**Table 2:** Generalized Linear Model fitted with Gamma distribution and log link depicting average CA density by Braak stage, controlling for age, PMI, APOE allele, cognitive status, and sex

Characteristic	Ratio of means	95% CI^a^	*p*-value
*Braak stage (I–VI)*			0.006
I	–	–	
II	1.72	0.73, 3.90	0.2
III	2.13	0.79, 5.69	0.11
IV	0.65	0.24, 1.70	0.4
V	0.52	0.19, 1.38	0.2
VI	0.64	0.25, 1.52	0.3
Age (years)	1.01	0.99, 1.03	0.3
PMI (minutes)	1.00	1.00, 1.00	0.4
*APOE allele (grouped)*			0.13
E23/E33	–	–	
E34/E44	0.67	0.41, 1.13	0.13
*Cognitive status*			0.4
Cognitively normal	–	–	
Mild impairment	1.70	0.64, 5.28	0.3
Dementia	1.59	0.80, 3.15	0.2
*Sex*			> 0.9
F	–	–	
M	1.01	0.64, 1.59	> 0.9

^a^CI = Confidence interval

**Table 3:** Multiple comparisons test of CA/mm^2^ by Braak Stage using Bonferroni method encompassing all hippocampal patient tissues analyzed in this study

Comparison	Adjusted *p*-value
II vs I	1.000
III vs I	1.000
IV vs I	1.000
V vs I	1.000
VI vs I	1.000
III vs II	1.000
IV vs II	0.267
V vs II	0.082
VI vs II	0.211
IV vs III	0.105
V vs III	0.034
VI vs III	0.106
V vs IV	1.000
VI vs IV	1.000
VI vs IV	1.000

### CA dynamics at specific Braak stages

**Fig. 3:**
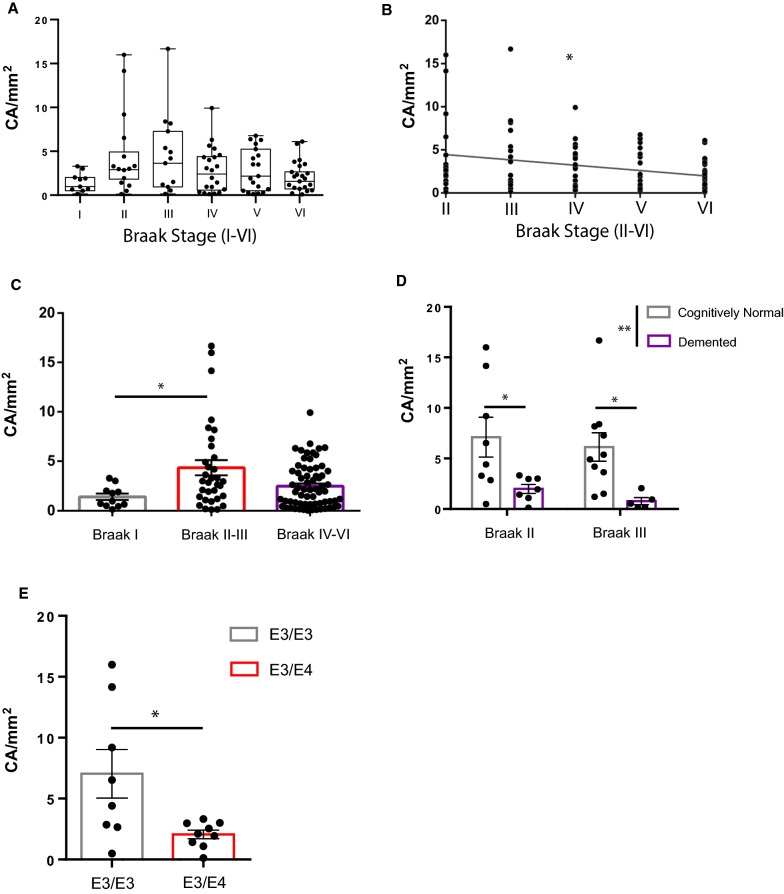
CA dynamics at specific Braak stages. **A** Box plot of DG CA/mm^2^ distributions across Braak stages I–VI with statistically-defined outliers excluded. **B** Scatter plot of DG CA/mm^2^ distributions across Braak stages II-VI with statistically-defined outliers excluded. Spearman r =−0.2164, *p = 0.0297. **C** Plot of DG CA/mm^2^ distributions pooled into early stage (Braak I), mid-stage (Braak II, III), and late-stage (Braak IV-VI) AD. *p = 0.0280, Kruskal–Wallis test. **D** Plot comparison of CA/mm^2^ in cognitively normal and demented patients at Braak II and Braak III. Two-way ANOVA, Sidaks multiple comparisons test. **p = 0.0016. *p = 0.033 (Braak II), 0.021 (Braak III). **E** Plot comparison of CA/mm^2^ in *APOE* E3/E4 single allele carriers (labeled as E3/E4) compared to *APOE* E3/E3 (labeled as E3/E3) at Braak II. *p = 0.0198, student’s t-test

Given that our generalized linear model detected a significant influence of Braak stage on CA levels with peak accumulation at Braak II–III, we sub-stratified our analysis based on Braak II as a critical threshold for CA abundance peaks. Using a refined cohort, in which we omitted statistically defined outliers determined by Grubb’s test (Additional file 3: Fig. S2, Q = 0.05), 112/124 cases were re-analyzed for CA at specific Braak stages (Fig. 3A). By focusing on Braak stages with tau deposition in the hippocampus (II-VI), Spearman’s nonparametric correlation reproduced the negative relationship between CA and Braak stage (Fig. 3B, r = −0.2164, *p = 0.0297). Multiple comparisons among Braak subgroups revealed significant hippocampal CA differences when comparing Braak I vs. II–III vs. IV–VI (Fig. 3C, *p* = 0.0280). These distributions suggest a critical window in which CA levels dynamically change in the DG; early CA accumulation parallels the appearance of tau pathology in Braak II–III, followed by CA decline at Braak IV–VI as tau burden accumulates in the hippocampus and propagates to cortical and neocortical brain regions [37–39].

### CA depletion in cognitively impaired and *APOE* E4 patients

We next examined whether the presence of both amyloid and tau pathology correlated with CA using a composite score of AD neuropathological change on a 0–3 scale (0 = none, 1 = mild, 2 = moderate, 3 = severe) [40]. Spearman correlation of CA levels with AD neuropathological score yielded a significant negative correlation (Additional file 4: Fig. S3A, r = −0.2728, *p* = 0.0046), suggesting that combined tau and amyloid co-pathology are associated with declining CA within the DG. We next compared CA levels between cognitively normal (CN) and dementia (DM) patients within the critical Braak II–III window, a point at which CA levels peak. We note that this analysis employed an adequately powered sample size of both CN and DM patients at Braak II–III. Within this sample group, we observed significantly reduced CA abundance in DM patients using two-way ANOVA and multiple comparison tests (Fig. 3D). We also compared CA levels in CN vs. DM patients at Braak IV, however no significant differences were observed (Additional file 4: Fig. S3B).

Given the above findings in APOE-KI mice that APOE status influences PAS granule formation (Fig. 1E–G), combined with previous reports that *APOE* E4 homozygotes display increased hippocampal atrophy [41], we next sought to determine if the presence of a single *APOE* E4 allele impacts CA levels in AD patient brain. A recent study of patients exposed to severe air pollution found increased CA in young and middle-aged *APOE* E4 carriers compared to *APOE* E3 controls [42]. Although our generalized model did not detect a significant influence of APOE allele on CA across all six Braak stages, we revisited the effect of APOE allele status using a stage-specific analysis. Using our refined patient cohort, we tested for gene dosage effect at Braak II and found that *APOE* E3/E4 carriers are associated with significantly lower CA compared to *APOE* E3/E3 carriers (Fig. 3E). No other significant differences in CA with respect to APOE status were detected at other Braak stages (Additional file 4: Fig. S3C–D). These data indicate that APOE allele variants may be linked to CA accumulation during early stages of tau deposition.

### Tau-immunoreactive CA are extruded into CSF

**Fig. 4:**
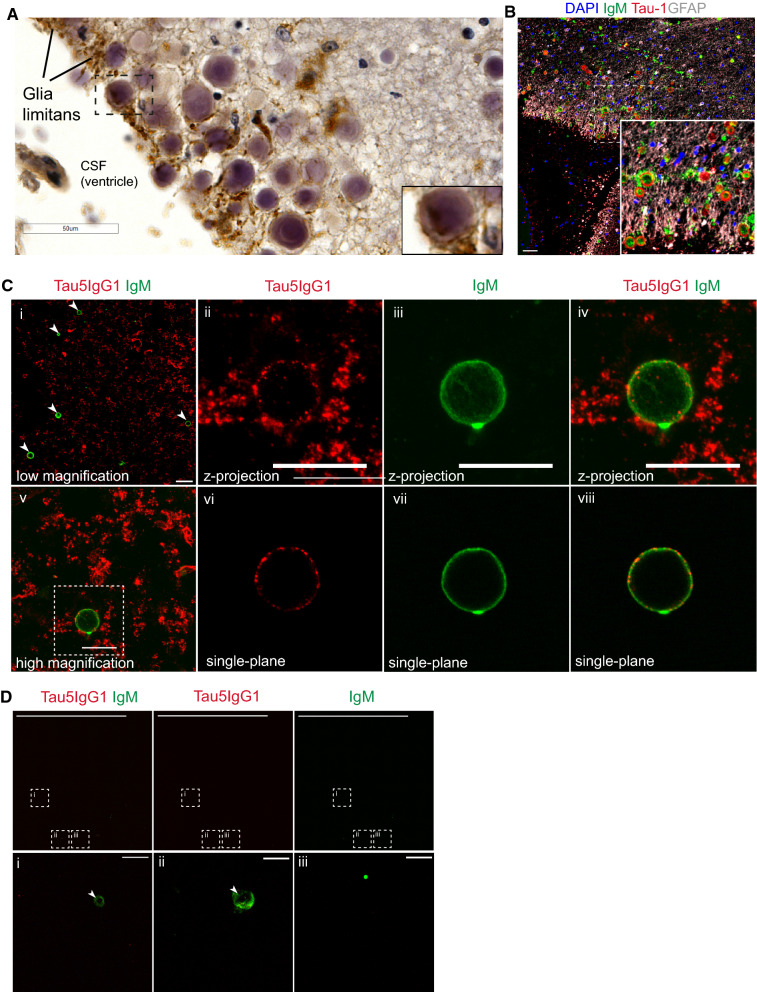
CA-tau structures are present in human CSF. **A** Representative brightfield image of CA in the hippocampal sulcus of AD patient tissue section. Inset (dashed box) centered on a CA in the glia limitans ensheathed in AT8^+^ processes on the edge of the parenchyma. Scale bar = 50 µm. **B** Representative immunofluorescent confocal image of CA in the hippocampal sulcus of AD patient tissue section stained with GFAP (grey), Tau-1 (red), and IgM (green). Nuclei stained with DAPI (blue). Inset (dashed box) centered on Tau-1-immunoreactive CA in the glia limitans ensheathed in GFAP^+^ processes on the edge of the parenchyma. Scale bar = 50 µm. **C** Representative immunofluorescent confocal images of CSF containing CA were stained with Tau5 IgG1 (red) and IgM (green) and subsequently imaged at 120× magnification. Scale bar = 50 µm (i) 20 µm (ii–v). Maximum intensity z-projection frames are shown in panels i and v, higher magnification inset frames are shown in panels ii-iv, and single plane inset frames are shown in panels vi–viii. **D** Representative immunofluorescent confocal images of CA present in CSF that were pre-digested with amylo-glucosidase were imaged at 20× magnification to detect Tau5 IgG1 (red) and IgM (green, brightness increased for visualization). Scale bar = 500 µm (top row), 10 µm (bottom row)

CA harbor Tau-1 (hypo-phosphorylated tau) and Tau5 (total tau) immunoreactive tau species in the brain that we previously referred to as CA-tau [17]. Prior work suggests that CA are extruded into the CSF before eventual passage to the lymph nodes [16]. Therefore, we sought to determine if the CA-tau identified in the brain becomes extruded into CSF. We first evaluated CA in proximity to the hippocampal sulcus and nearby ventricles. Interestingly, in late Braak samples, CA were often enveloped by AT8-positive tau pathology at the glia limitans, the outer surface of the brain parenchyma (Fig. 4A). Indeed, we observed co-localization of CA (IgM reactive) with markers of hypo-phosphorylated tau (Tau-1) by confocal imaging (Fig. 4B). Next, we qualitatively surveyed the presence of CA in CSF samples from healthy control and AD patients. We enriched for CA using a low-speed differential centrifugation protocol followed by resuspension of CA-containing pellets, fixation on slides, and immunostaining with Tau5 and IgM primary antibodies, and in one control patient sample observed numerous instances of Tau5^+^ CA (Fig. 4C). As negative controls, CSF-enriched CA were not detected in the absence of primary IgM antibodies (Additional file 5: Fig. S4). Similarly, CSF-enriched CA were depleted by enzymatic digestion with amyloglucosidase, which removes IgM-reactive carbohydrate moieties on the CA surface [14, 43]. In these digested fractions, we observed partially formed CA that were weakly IgM-immunoreactive (Fig. 4D). CSF-enriched CA were not detected by microscopy in any AD patients within our cohort. These findings suggest that CA-tau is detectable in human CSF and could provide diagnostic or prognostic utility as a potential tauopathy biomarker, though we emphasize that larger CSF sample cohorts (from AD and other tauopathies) will be required for future quantitative biomarker studies.

## Discussion

This study establishes a link between PAS granules and CA with known hallmarks of AD, namely tau deposition and symptomatic progression of AD. Our prior study identified hippocampal granules (that we found to be synonymous with PAS granules) present in aged and diseased mouse models [9, 30]. While several APP-overexpressing mouse models have now been shown to harbor PAS granules in the molecular layer of the hippocampus [24, 44], studies had not been conducted in tauopathy models, in which more severe cognitive decline and neurodegeneration is frequently observed.

**Fig. 5:**
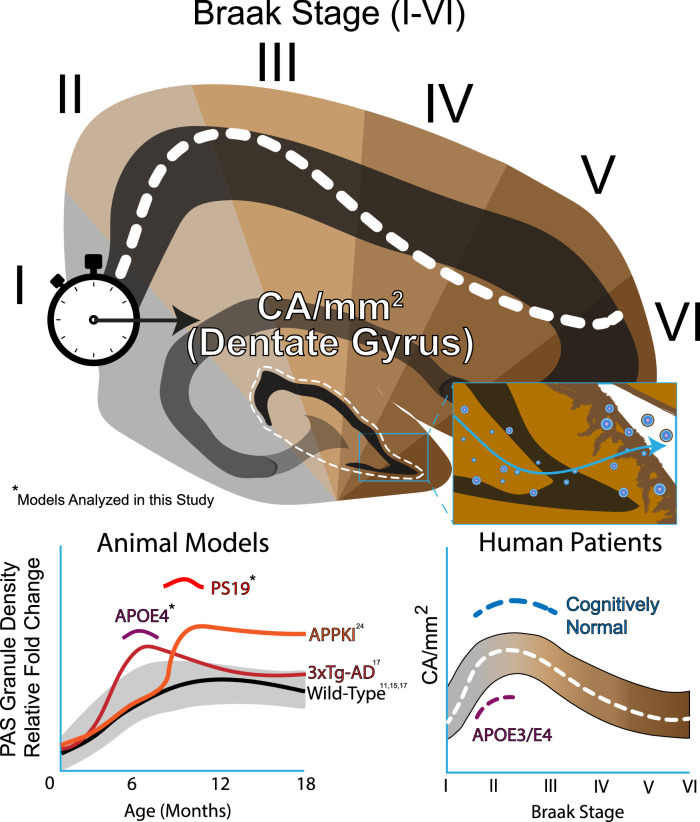
Dentate CA depletion is associated with *APOE* E4 status and cognitive decline. Schematic describing correlation between Braak stage and CA decline in the DG. CA accumulate in the dentate gyrus hilus proximal to the hippocampal sulcus, where they are extruded via the glia limitans. CA harbor Tau-1-immunoreactive hypophosphorylated (not hyperphosphorylated) tau that may be released into the CSF. In both mouse models of AD risk and tauopathy, PAS granule density is elevated (asterisks denote single time-point assessments of PAS granule density from this study). In humans, CA levels (as determined by CA/mm^2^) are reduced in dementia patients and *APOE* E4 carriers at critical tau-seeding Braak stages when compared to cognitively normal or *APOE* E4 non-carriers, respectively. Our model is consistent with the hypothesis that CA are protective against the accumulation of AD pathology but become exhausted with increasing neuropathological burden in human brain

Our analysis of PS19 tauopathy mice showed elevated, not depleted, PAS granule density at advanced stages of tau accumulation, which we note contrasts with the trajectory of human CA at comparable stages of hippocampal tau deposition. We also observed an increase in PAS granule density within the hippocampus of *APOE* E4 knock-in mice when compared to age-matched *APOE* E3 controls in the absence of tau pathology or neurodegeneration. PAS granules and CA are closely associated with astrocytes [45] and, to a lesser extent microglia [17], both of which are implicated in tau-mediated neurodegeneration [46, 47]. These results are consistent with PAS granule formation as a compensatory stress response in a wide spectrum of mouse models showing phenotypes of accelerated aging (SAMP), inflammation (LPS), and amyloid accumulation [11, 12, 24]. It is currently unclear why PS19 mice show elevated PAS granule formation, while their human counterpart (CA) become gradually depleted with advancing Braak stage. Importantly, there are key differences in the stress response between human and murine astrocytes [48]. In addition, human AD brain represents a synergy resulting from multiple co-pathologies that may not be adequately recapitulated in individual APP, tau, or APOE mouse models, which could impact PAS granule/CA dynamics [49, 50]. Supporting this latter possibility, 3xTg mice harboring both amyloid and tau show a peak PAS granule density at 10 month old, which slowly tapered off until 24 months [17]. These findings suggest that factors accelerating disease progression (e.g., tau seeding and propagation) could further lead to a gradual decline in CA proteostasis (Fig. 5).

Using a panel of human AD brains, we found that Braak II and Braak III patients exhibited the highest CA burden in the DG, which then gradually diminished at later Braak stages. Braak II is characterized by the propagation of AT8^+^ tau lesions into the entorhinal regions and is the first stage during which tau lesions accumulate in the hippocampus [51]. Thus, the peak CA threshold coincides with the first pathological manifestations of tau in the hippocampus, aligning with findings that tau distribution is a strong indicator of regional atrophy [52]. Our findings suggest that failed waste clearance is associated with CA dysfunction, an effect that is accelerated by the presence of a single *APOE* E4 allele (Fig. 3E) [53]. This is consistent with previous observations of elevated CA in the olfactory bulb of young and middle-aged human *APOE* E4 carriers exposed to environmental insult via air pollution [42], and with our findings that CA are reduced in a subset of demented *APOE* E4 individuals at Braak stage II (the peak of CA density). If CA do indeed act as a compensatory protective response to tau in AD, one could predict that their accumulation signifies a stress response that coincides with neuronal damage (Fig. 5). Beyond tau, CA are likely responsive to other perturbations within the brain, likely acting generally to eliminate aggregated proteins, damaged organelles and other factors [4, 18], and possibly microbial elements [18, 56], implying broad relevance to neurological diseases [1, 14, 57, 58]. Indeed, CA accumulation or a related process, astrocytic clasmatodendrosis, may occur in brains from younger patients exposed to drug-induced stress [54, 55].

Like PAS granules, CA formation is thought to be central to astrocytes, a prominent cell type in pial and subpial zones proximal to the glia limitans [16, 59], where we note the accumulation of AT8^+^ structures in late-stage AD patients (Fig. 4A and Table 4). Several studies have noted CA in close proximity to aging-related tau astrogliopathy (ARTAG) [60, 61], characterized by AT8^+^ aggregated tau in soma and processes of astrocytes with diverse morphological characteristics [62, 63]. Future side-by-side comparisons using epitope and biochemical profiling of CA-tau and glial tau in ARTAG could shed light on the extent to which these two phenomena are interrelated.

**Table 4:** Extended human sample characteristics from cases used in this study

Characteristic	N = 124
*Dentate gyrus area (mm*^*2*^*)*	
Mean (SD)	9.4 (3.8)
Median (IQR)	8.9 (7.1, 11.3)
Range	2.4, 24.0
*PMI (minutes)*	
Mean (SD)	770 (570)
Median (IQR)	680 (296, 1,068)
Range	30, 2,911
Not recorded	10
*AD neuropathological score*	
None	14 (14%)
Mild	18 (18%)
Moderate	44 (45%)
Severe	22 (22%)
Not recorded	26
*Amyloid angiopathy*	
None	50 (40%)
Mild	40 (32%)
Moderate	25 (20%)
Severe	9 (7.3%)
*Atherosclerosis*	
None	70 (56%)
Mild	26 (21%)
Moderate	21 (17%)
Severe	7 (5.6%)
*Arteriosclerosis*	
None	86 (69%)
Mild	13 (10%)
Moderate	19 (15%)
Severe	6 (4.8%)
*Hippocampal sclerosis (0 = not observed/recorded, 1 = present)*	
0	112 (90%)
1	12 (9.7%)
*McKeith DLB (0 = not observed/recorded, 1 = present)*	
0	96 (77%)
Amygdala	4 (3.2%)
Brainstem	2 (1.6%)
Limbic	4 (3.2%)
Neocortex	18 (15%)
*Infarction (0 = not observed/recorded, 1 = present)*	
0	107 (86%)
1	17 (14%)
*Hemorrhage (0 = not observed/recorded, 1 = present)*	
0	119 (96%)
1	5 (4.0%)
*ARTAG (0 = not observed/recorded, 1 = mild ARTAG)*	
0	113 (91%)
1	11 (8.9%)
*LATE: Limbic-PAR TDP-43 (0 = not observed/recorded, 1 = present)*	
0	114 (92%)
1	10 (8.1%)
*A Score*	
0	11 (11%)
1	18 (18%)
2	9 (9.1%)
3	61 (62%)
Not recorded	25
*A Score (Thal)*	
0	11 (11%)
1	12 (12%)
2	6 (6.1%)
3	9 (9.1%)
4	37 (37%)
5	24 (24%)
Not recorded	25
*B Score (Braak)*	
1	27 (27%)
2	35 (35%)
3	37 (37%)
Not recorded	25
*C Score (CERAD)*	
0	23 (23%)
1	19 (19%)
2	35 (36%)
3	21 (21%)
Not recorded	26

Overall, our study provides evidence that CA depletion in AD is associated with key correlates of disease including advancing tau burden, APOE status, and cognition in AD patients. To determine the utility or feasibility of CA as biomarkers or therapeutic targets, future efforts are needed to delineate the mechanisms surrounding their generation, encapsulation of tau cargo, and clearance. Rather than acting as a generic neuropathological hallmark of aging, these findings ascribe new disease relevance to CA, and support the development of quantitative methods to detect CA in human brain and CSF as a biomarker for AD, related tauopathies, and perhaps other neurodegenerative diseases.

## Materials and methods

### Immunofluorescence staining (IF)—human tissue

For all qualitative IF analysis of human brain tissue samples, freshly cut sections were prepared from late stage (Braak V–VI) AD and control patient tissues archived at the UNC Brain Bank. Paraffin embedded tissue blocks were sectioned at 7 µM thickness, adhered to slides, and deparaffinized in xylene. Slides were rinsed in ethanol and rehydrated (pure, 95%, 70%) in 1 X PBS. Sections were boiled for 10 min and incubated in hot Vectastain H-3300 citrate-based buffer for 30 min for antigen retrieval. Sections were then rinsed in 1X TBS, and permeabilized for 50 min in 2% TBS-Triton X-100 at RT. Blocking, primary, and secondary solutions matched the donor species of secondary antibodies used; sections were blocked in 1X TBS with 2% goat serum for 50 min. Sections were then incubated with primary antibody in 0.1% sodium azide solution for 48 h at RT, thoroughly rinsed in 1X TBS three times, and incubated in secondary antibody solution for 24 h at RT. DAPI staining was used to visualize nuclei. Primary antibodies and dilutions: Tau-1 (Millipore, MAB3420, 1:1000), GFAP 1:1000, (Dako, GA524). Secondary antibody dilutions: Alexa Fluor goat anti-mouse IgM μ-chain specific 488, 1:200 (Thermofisher, A-21042), Alexa Fluor goat anti-rabbit 568, 1:200 (Thermofisher, A-11011), Alexa Fluor goat anti-mouse IgG1 specific 647, 1:200 (Thermofisher, A-1240).

### Immunofluorescence Staining (IF)—mouse tissue

Mice were deeply anesthetized and transcardially perfused with 1X PBS and 15 mL 4% Paraformaldehyde. Brains were then post-fixed for 24–48 h in 4% PFA at 4 °C and cryoprotected in 30% sucrose solution before sectioning. Tissues were sectioned at 30–40 μM thickness and stored at −20 °C. Immunofluorescence staining was conducted using a free-floating technique, rinsing in 1X TBS between all major steps, gently agitated on a plate shaker. Sections were rinsed and permeabilized for 50 min in 2% TBS-Triton X-100 at RT. Blocking, primary, and secondary solutions matched the donor species of secondary antibodies used; sections were blocked in 1X TBS with 2% goat serum for 50 min each. Sections were then incubated with primary antibody in 0.1% sodium azide solution for 48 h at room temperature, thoroughly rinsed, and incubated in secondary antibody solution for 24 h at room temperature. DAPI staining was used to visualize nuclei. Primary antibodies and dilutions: Mouse IgM, kappa monoclonal MM-30, 1:1000 (Abcam, ab18401), AT8 1:500 (Thermofisher, MN1020), GFAP 1:1000, (Dako, GA524). Secondary antibodies and dilutions: Alexa Fluor goat anti-mouse IgM μ-chain specific 488, 1:200 (Thermofisher, A-21042), Alexa Fluor goat anti-rabbit 568 1:200 (Thermofisher, A-11011), Alexa Fluor goat anti-mouse IgG1 specific 647, 1:200 (Thermofisher, A-21240).

### Immunohistochemical Staining (IHC)—Human tissue

For all IHC analysis of formalin fixed, paraffin embedded tissues (*n* = 124 total cases), brain samples were acquired from the Duke Bryan Brain Bank (Table 1), and samples were sectioned at 8 µm thickness and adhered to slides. Tissue sections were deparaffinized with xylene and rinsed with ethanol (pure, 95%) prior to staining. Endogenous peroxidase activity was blocked via incubation in 1.875% H2O2 in methanol for 8 min. Sections were rinsed with 1X PBS, and blocked with 5% non-fat dry milk in 0.05 M Tris buffer, Ph7.6 for 20 min at room temperature. Sections were rinsed in deionized water three times for two minutes each. Sections were incubated for 45 min at 37 °C with AT8 (Thermofisher, MN1020, 1:500) primary antibody, rinsed three times in deionized water, and incubated for 30 min with Dako EnVision Dual Link System-HRP at 37 °C. Sections were again rinsed in deionized water three times before development with Dako DAB Solution (Agilent, K346811-2) for 5 min before rinsing in tap water for 5 min. Counterstain was applied with Fisherfinest Hematoxylin + (220-100) for 25 s before an additional tap water rinse for 5 min. Blueing of sections was achieved with ammoniacal water for 10 s before a final 3-min rinse in tap water. Sections were dehydrated in serial graded ethanol rinses, cleared in xylene and coverslipped with permount before scanning and analysis.

### Analysis of IHC sections

Neuropathological evaluations were performed by the Duke Bryan Brain Bank, including Braak stage, AD composite neuropathological score, age, postmortem interval, cognitive status, sex, ethnicity, and APOE allele status (Table 1). Composite neuropathological scoring was assessed according to guidelines in Montine et al. [40]. Tissue sections were immunostained with AT8 for tau pathological staging as previously described [64] and counterstained with hematoxylin to identify CA structures. All slides were scanned at the UNC Translational Pathology Laboratories on a brightfield Scanscope AT2 at 40× magnification. Images were inspected for quality and cropped to the hippocampal region in Aperio Imagescope; within this image, the dentate gyrus was traced as the analysis region of interest, and area measured for CA density calculation. CA were annotated and counted by three independent investigators blinded to patient status. To distinguish CA from smaller nuclei within regions of interest, criterion for CA detection included low to moderate hematoxylin staining intensity with a uniform or concentric ring pattern and circularity.

### Statistical analysis—PAS granules in tauopathy and APOE-KI mice

The WT and PS19 animals were compared in terms of PAS granule density, GFAP Intensity, and AT8 intensity using an unpaired t-test, with a 5% significance level. The overall difference in PAS granule density between APOE-KI mice (E2, E3, E4) was assessed using an unpaired t-test, with 5% significance level. Stratified analysis by sex was also performed.

### Statistical analysis—CA quantification

Summary statistics including mean, standard deviation, median, interquartile range, and range were computed for the continuous variables. Absolute and relative frequencies were calculated for the categorical variables. Box plots and scatter plots were constructed to assess the association between CA and Braak stage. Plots using the log scale for the CA counts are also provided. The association between CA and Braak stage was then evaluated using a generalized linear model with gamma distribution and log link function, controlling for age (years), PMI (minutes), cognitive status, sex, and APOE allele. Results are presented as mean ratios (MR) with confidence intervals (CI). Multiple comparisons among Braak stages are performed using the Bonferroni method. All statistical analyses were performed in R version 4.0.2 (R Core Team, 2020). Complete case analysis was considered, with *P* < 0.05 determining statistical significance.

### Immunofluorescence staining (IF)—human CSF

For qualitative IF analysis of CA present in CSF, human control and AD patient CSF samples (n = 2 patient samples for each) were obtained from the University of Miami Brain Bank, aliquoted, and stored at -80 °C. Aliquots (200 μl each) were centrifuged three times for 10 min at 700 g in 4 °C before fractionation for amyloglucosidase digestion and resuspended with 1X PBS each time. Digested fractions were incubated with 10U amyloglucosidase (Sigma, 9032-08-0) in phthalate buffer (pH 5) for 24 h at 45 °C. Undigested fractions were incubated at 4 °C for 24 h in phthalate buffer without amyloglucosidase. After digestion/incubation, fractions were centrifuged three times for 10 min at 700 g in 4 °C and resuspended with 1X PBS each time. Following the final centrifugation, samples were extended on charged Superfrost slides, air dried overnight, and fixed with acetone for 10 min at 4 °C. Slides were then rinsed with 1X TBS three times for 5 min, before being permeabilized with 1X TBS + 2% Triton X-100 for 30 min at RT. Slides were incubated with blocking buffer (1X TBS + 2% Goat Serum) for 30 min at RT. Digested and undigested fractions were incubated overnight at RT in blocking buffer containing Tau5 (Ms IgG1, MAB361, Millipore) at 1:200. Secondary only controls were instead incubated in empty blocking buffer. All samples were then incubated overnight at RT in secondary antibody solution (blocking buffer) with Alexa-Fluor anti-mouse IgM 488 (Thermo, A-21042) at 1:200 and Alexa-Fluor anti-mouse IgG1 (Thermo, Cat A-21240) at 1:200. Slides were coverslipped in Fluoromount-G and dried overnight before imaging at 60× magnification on an Olympus Fluoview FV3000RS Confocal laser scanning microscope. Since this was a qualitative analysis performed by immunofluorescence microscopy (representative images are depicted in Fig. 4C), additional sample numbers will be required for future statistical comparisons between sample groups (e.g., control vs. AD).

### Microscopy

Confocal images were captured on an Olympus FV3000RS microscope using resonant, one-way scanning. The following fluorophores were used: Alexa Fluor 405 (DAPI), Alexa Fluor 488, Alexa Fluor 568, Alexa Fluor 594, Alexa Fluor 647. Laser intensities, gains, and offsets were set at thresholds that reflect minimal fluorescence in respective control stains of sections incubated with only blocking buffer and secondary antibodies for each experiment. Imaging parameters were tailored for each experimental application, but were kept consistent between genotypes, adsorption pairs, and all other respective experimental and control stains; sections used for quantification of PAS granules were imaged at 20× magnification with 2 × zoom. For co-localization analysis, human tissue sections were imaged at 20× magnification with 3 × zoom. Phase separation ensured that fluorescence spectra did not significantly overlap (pairing: 405 + 568/594, 488 + 647, when applicable). High-resolution images for analysis of CA in CSF were captured using 60× magnification and 2 × zoom.

## Supplementary Information

**Additional file 1**:** Figure S1**. CA analysis of AD cases spanning Braak stages I–VI. **A** Representative brightfield images of full dentate gyrus architecture in patient tissue samples from Braak I–VI. Inset (dashed box) indicates sample region used in Fig. 2A. Scale bars = 900 µm (I, IV), 500 (III) 400 µm (V), 600 µm (II, VI).

**Additional file 2**: ** Figure S1**. **B** CA/mm^2^ distribution plot for all 124 patients included in this study showing a skewed distribution of patient CA/mm^2^. **C** Logarithmic distribution plot of CA/mm^2^ for all 124 included in this study. **D** Box plot of CA/mm^2^ vs Braak Stage using all 124 patients included in this study. **E** Logarithmic box plot of CA/mm^2^ vs Braak Stage (I–VI) displaying all 124 patients included in this study.

**Additional file 3**:** Figure S2**. CA analysis of a refined AD cohort lacking outliers. Scatter plot of refined patient cohort, CA Density vs Braak Stage I, II, III, IV, V, and VI. (112 patients in black, 12 outliers identified by Grubb’s test, Q = 0.05, excluded patients in red). Statistically identified outliers are depicted in the table. Pathological and demographic information for outliers excluded from analysis, sorted by Braak stage. From left to right: Alzheimer’s neuropathological score, age, APOE allele, cognitive status, post-mortem interval, sex, dentate gyrus area, CA numerical count, and CA/mm^2^.

**Additional file 4**:** Figure S3**. CA analysis in a refined AD cohort illustrates a correlation with cognition and APOE status. **A** Scatter plot of CA/mm^2^ vs AD Neuropathology score, ranked 0–3 (0 = none, 1 = mild, 2 = moderate, 3 = severe) for 112 patients, 12 outliers excluded. Spearman r = -−0.2728, **p = 0.0046. **B** Plot of CA/mm^2^ of cognitively normal and demented patients at Braak Stages II, III, and IV. (112 patients, 12 outliers excluded). * p = 0.033 (Braak II), 0.021 (Braak III). **C** Plot of CA/mm^2^ in E3/E3 vs E3/E4 patients at Braak Stages I, II, III, IV, and V. (112 patients, 12 outliers excluded)**.** *p = 0.0198. **D** Plot of CA/mm^2^ in E3/E3 vs E3/E4 and E4/E4 patients at Braak stages IV, V and VI. (112 patients, 12 outliers excluded).

**Additional file 5**:** Figure S4**. The detection of human CA in CSF requires primary IgM antibodies. **A** Shown is a secondary only control stain for immunofluorescent detection of CA in human patient CSF. No CA are detected in the absence of primary IgM antibody, though we note the detection of some non-specific debris. AlexaFluor anti-Ms IgG1 (red) and Anti-Ms IgM (green, brightness increased for visualization). Scale bar = 100 µm.  Bottom row frames are insets from top row frames within the dashed white compartment.

## Data Availability

Original slides and diagnostic material are retained at University of North Carolina and Duke University. Mouse slides and tissues are available upon request. All other materials are commercially available.
