# Supplementary figures and images for: Corpora amylacea are associated with tau burden and cognitive status in Alzheimer’s disease
Source: Acta Neuropathol Commun. 2022 Aug 8;10:110. doi: 10.1186/s40478-022-01409-5 (PMC9361643; doi:10.1186/s40478-022-01409-5)

A

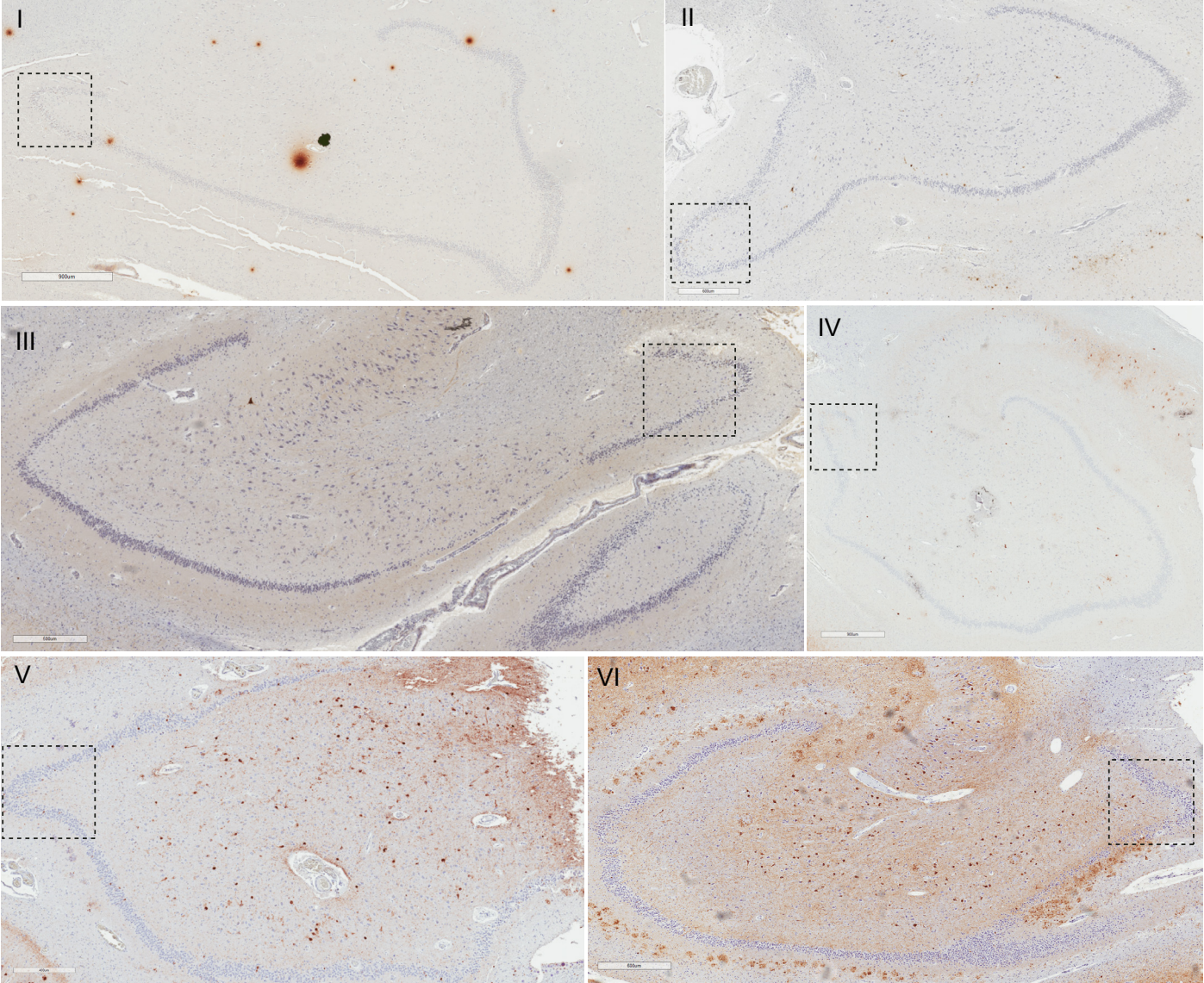

Supplement: Supplementary file 1 — Additional file 1: Figure S1. CA analysis of AD cases spanning Braak stages I–VI. A Representative brightfield images of full dentate gyrus architecture in patient tissue samples from Braak I–VI. Inset (dashed box) indicates sample region used in Fig. 2A. Scale bars = 900 µm (I, IV), 500 (III) 400 µm (V), 600 µm (II, VI). [file 40478_2022_1409_MOESM1_ESM.pdf]

**B**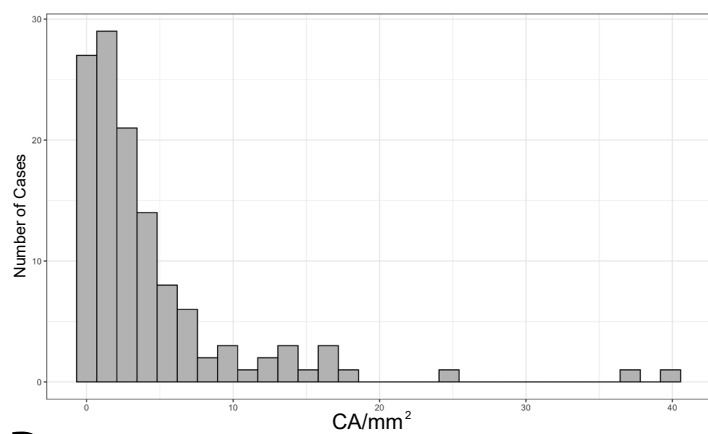**C**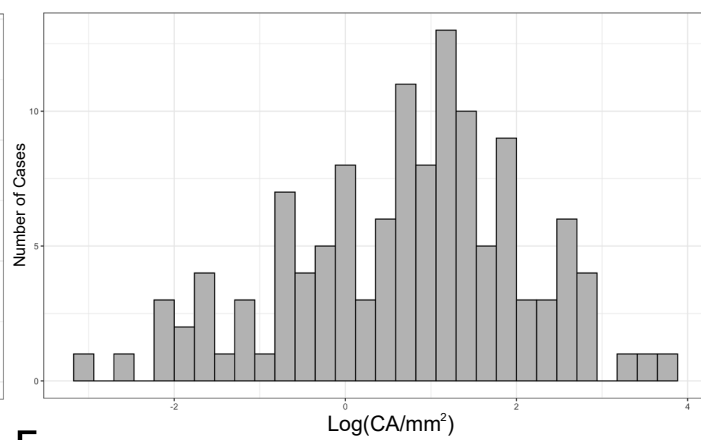**D**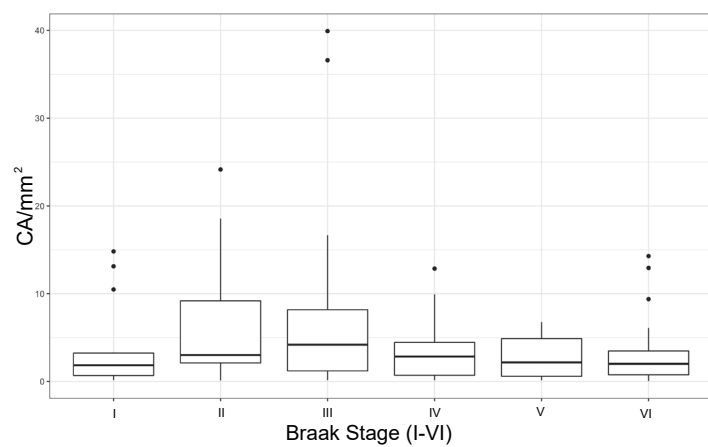**E**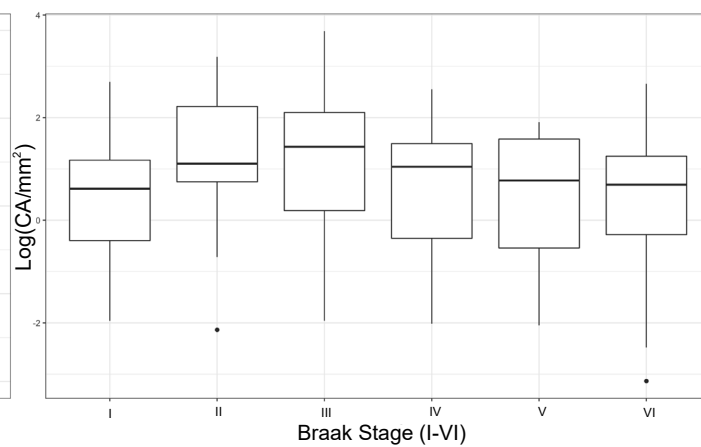

Supplement: Supplementary file 2 — Additional file 2: Figure S1. B CA/mm2 distribution plot for all 124 patients included in this study showing a skewed distribution of patient CA/mm2. C Logarithmic distribution plot of CA/mm2 for all 124 included in this study. D Box plot of CA/mm2 vs Braak Stage using all 124 patients included in this study. E Logarithmic box plot of CA/mm2 vs Braak Stage (I–VI) displaying all 124 patients included in this study. [file 40478_2022_1409_MOESM2_ESM.pdf]

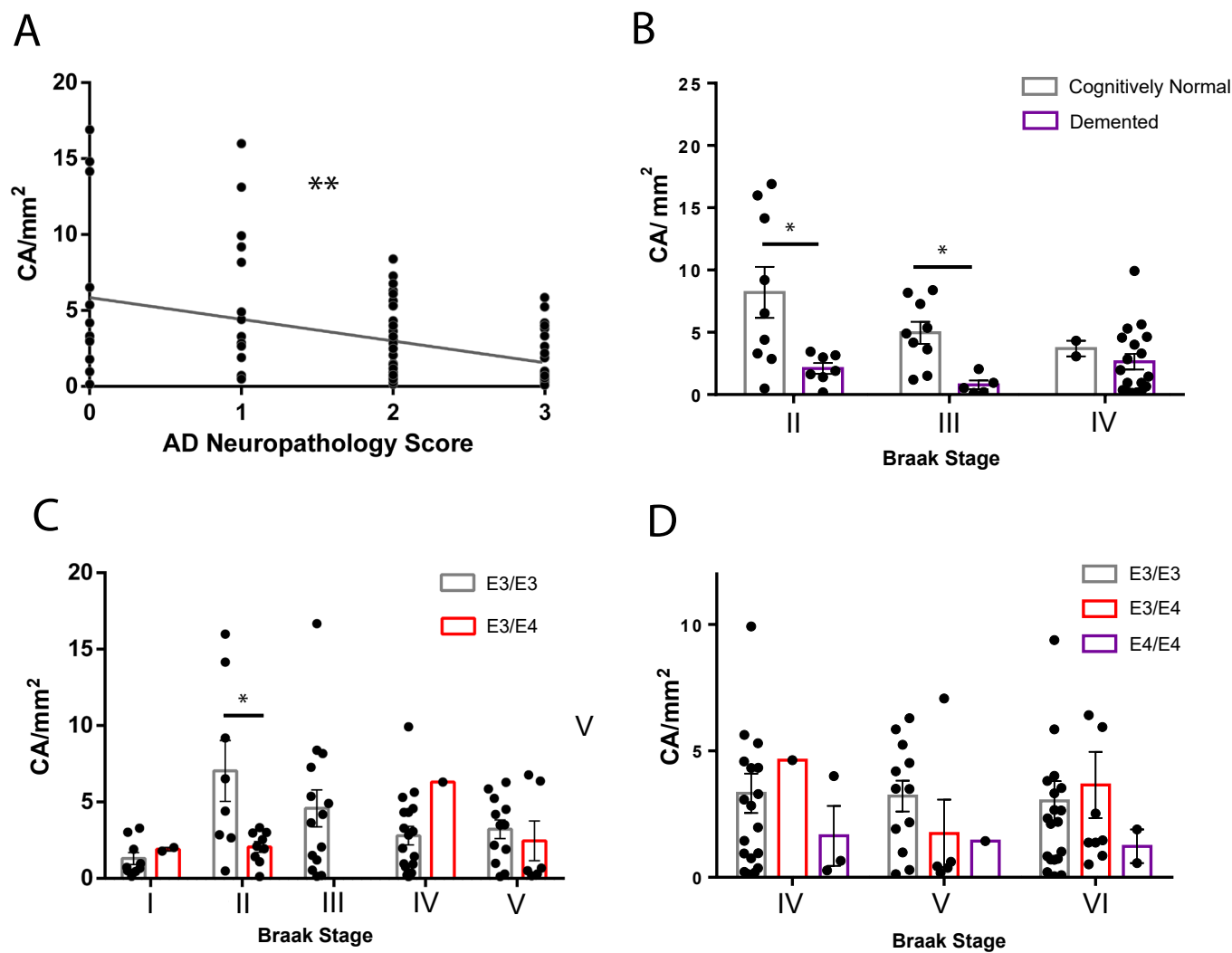

Supplement: Supplementary file 4 — Additional file 4: Figure S3. CA analysis in a refined AD cohort illustrates a correlation with cognition and APOE status. A Scatter plot of CA/mm2 vs AD Neuropathology score, ranked 0–3 (0 = none, 1 = mild, 2 = moderate, 3 = severe) for 112 patients, 12 outliers excluded. Spearman r = -−0.2728, **p = 0.0046. B Plot of CA/mm2 of cognitively normal and demented patients at Braak Stages II, III, and IV. (112 patients, 12 outliers excluded). * p = 0.033 (Braak II), 0.021 (Braak III). C Plot of CA/mm2 in E3/E3 vs E3/E4 patients at Braak Stages I, II, III, IV, and V. (112 patients, 12 outliers excluded). *p = 0.0198. D Plot of CA/mm2 in E3/E3 vs E3/E4 and E4/E4 patients at Braak stages IV, V and VI. (112 patients, 12 outliers excluded). [file 40478_2022_1409_MOESM4_ESM.pdf]

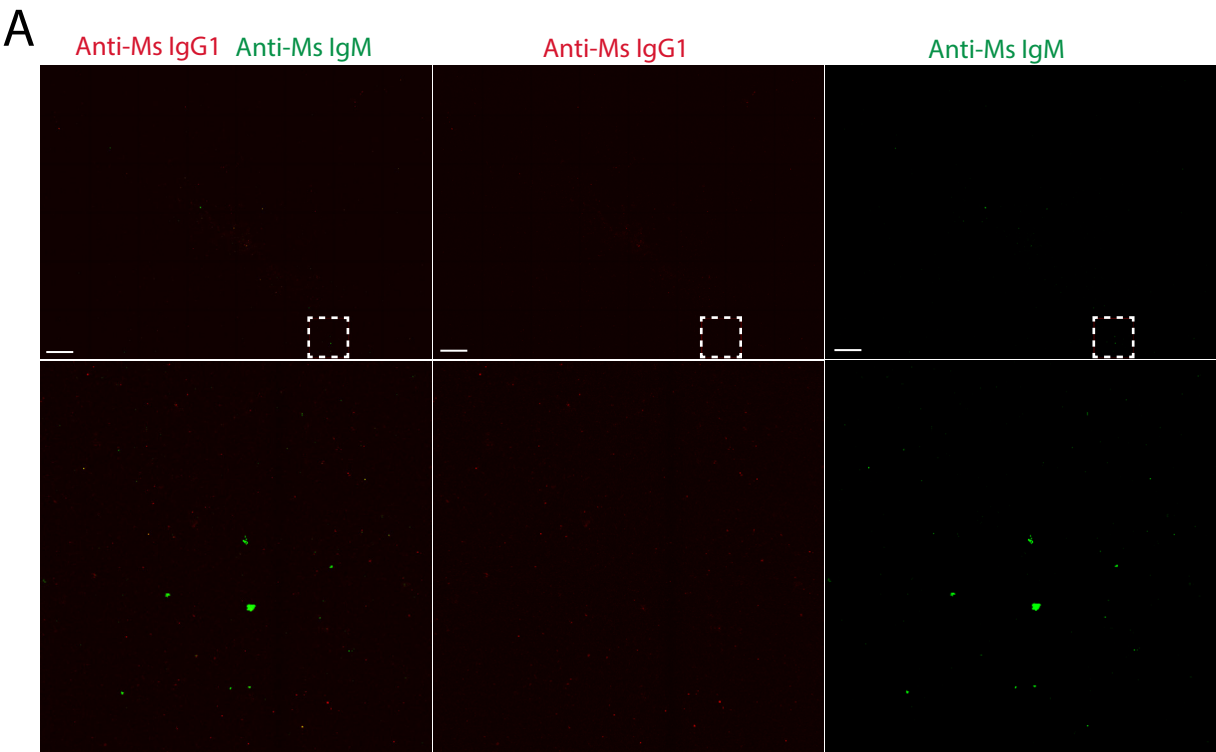

Supplement: Supplementary file 5 — Additional file 5: Figure S4. The detection of human CA in CSF requires primary IgM antibodies. A Shown is a secondary only control stain for immunofluorescent detection of CA in human patient CSF. No CA are detected in the absence of primary IgM antibody, though we note the detection of some non-specific debris. AlexaFluor anti-Ms IgG1 (red) and Anti-Ms IgM (green, brightness increased for visualization). Scale bar = 100 µm. Bottom row frames are insets from top row frames within the dashed white compartment. [file 40478_2022_1409_MOESM5_ESM.pdf]
