# Supplementary material for: Corpora amylacea are associated with tau burden and cognitive status in Alzheimer’s disease
Source: Acta Neuropathol Commun. 2022 Aug 8;10:110. doi: 10.1186/s40478-022-01409-5 (PMC9361643; doi:10.1186/s40478-022-01409-5)
Supplement: Supplementary file 3 — Additional file 3: Figure S2. CA analysis of a refined AD cohort lacking outliers. Scatter plot of refined patient cohort, CA Density vs Braak Stage I, II, III, IV, V, and VI. (112 patients in black, 12 outliers identified by Grubb’s test, Q = 0.05, excluded patients in red). Statistically identified outliers are depicted in the table. Pathological and demographic information for outliers excluded from analysis, sorted by Braak stage. From left to right: Alzheimer’s neuropathological score, age, APOE allele, cognitive status, post-mortem interval, sex, dentate gyrus area, CA numerical count, and CA/mm2. [file 40478_2022_1409_MOESM3_ESM.pdf]

Wander et al, Supplementary Figure 2

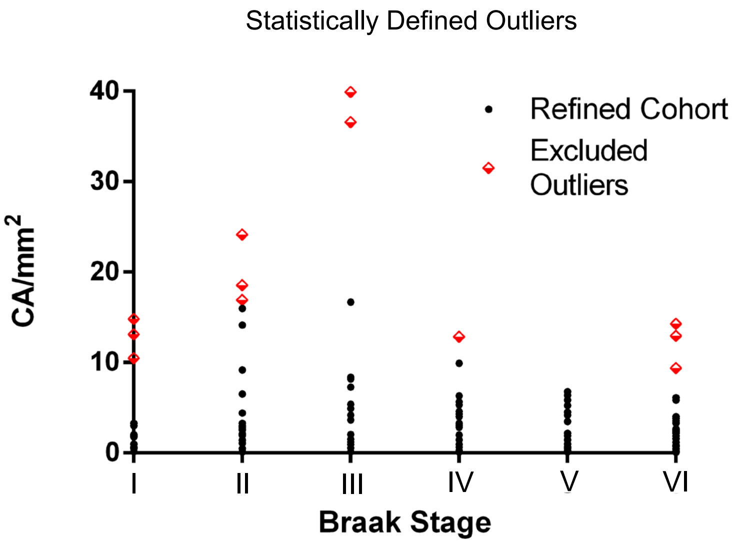

Braak Stage	AD Npath (0=none; 1 = mild; 2 = moderate; 3 = severe)	Age	APOE Allele	Cognitive Status	PMI (minutes)	Sex	DG Area	CA Number	Averaged CA/mm^2
I	0	62	E3/E3	Dementia	290	M	5827622.18	96	14.81598887
I	0	71	E3/E4	Cognitively Normal	225	F	10021620.48	107	10.48253363
I	1	>95	E3/E3	Mild Impairment	590	F	9275673.1	112	13.12099027
II	0	64	E3/E3	Cognitively Normal	640	F	11730930.79	184	16.90854839
II	NA	89	E3/E4	Dementia	30	M	7222001.36	178	24.1522476
II	1	90	E3/E3	Dementia	720	M	10212334.95	148	18.55620313
III	2	76	E3/E3	Dementia	630	F	2432495.33	166	39.91700288
III	2	77	E3/E3	Dementia	2070	M	4175098.58	146	36.59635402
IV	2	90	E3/E3	Mild Impairment	980	F	7448827.2	87	12.85581728
VI	3	59	E3/E3	Dementia	285	M	5806202.99	71	14.2854691
VI	NA	71	E3/E3	Dementia	1510	M	16229781.3	9	9.384647084
VI	2	84	E3/E4	Dementia	615	F	4359060.85	38	12.9347703
